# Analysis of gut microbiota of ladybug beetle (*Harmonia axyridis*) after feeding on different artificial diets

**DOI:** 10.1186/s12866-023-03155-7

**Published:** 2024-01-03

**Authors:** Bing-Hua Xie, Lei Chao, Si-Jing Wan, Hui-Ru Si, Wei-Dong Yu, Zhen Huang, Shi-Gui Wang, Nicolas Desneux, Bin Tang, Si-Si Sun

**Affiliations:** 1 College of Life and Environmental Sciences, Hangzhou Normal University, Hangzhou, 311121 Zhejiang China; 2 Zhejiang Dingyi Biotechnology Corporation, Quzhou, 324100 Zhejiang China; 3 UMR ISA, Université Côte d’Azur, INRAE, 06000 Nice, France; 4 Guizhou Institute of Mountainous Meteorological Sciences, Guiyang, 550002 Guizhou China

**Keywords:** Firmicutes, *Harmonia axyridis*, Proteobacteria, Synergistic substance

## Abstract

**Background:**

*Harmonia axyridis* is an effective natural enemy insect to a variety of phloem-sucking pests and *Lepidopteran* larvae, such as aphids, scabies, and phylloxera, while its industrial production is limited due to unmature artificial diet. Insect intestinal microbiota affect host development and reproduction. The aim of this study is to understand intestinal microbiota composition of *H. axyridis* and screen effective probiotics on artificial diet. Considering the role of the components and composition of the diet on the structure and composition of the intestinal microbiome, four kinds of diets were set up: (1) aphid; (2) basic diet; (3) basic diet + glucose; (4) basic diet + trehalose. The gut microbiota of *H. axyridis* was detected after feeding on different diets.

**Results:**

Results showed that the gut microbiota between artificial diet group and aphid groups were far apart, while the basic and glucose groups were clearly clustered. Besides, the glucose group and trehalose group had one unique phylum, *Cryptophyta* and *Candidatus Saccharibacteria*, respectively. The highest abundance of *Proteobacteria* was found in the aphid diet. The highest abundance of *Firmicutes* was found in the basic diet. However, the addition of glucose or trehalose alleviated the change. In addition, the relative abundance of *Enterobacter*, *Klebsiella*, *Enterobacteriaceae*_unclassified, *Enterobacteriales*_unclassified and *Serratia* in the aphid group was higher than other groups. Moreover, the function of gut genes in each group also showed clear differences.

**Conclusion:**

These results have offered a strong link between artificial diets and gut microbes, and also have provided a theoretical basis for the screening of synergistic probiotics in artificial diet.

## Background

Insects are one of the most diverse and oldest life forms on Earth, with a wide variety of species and a large population, which exist in almost all ecosystems on Earth [1, 2]. While climate warming affects crop phenology, physiology and biochemistry in agricultural ecosystems and has cascading effects on the performance and abundance of herbivorous insect pests [3, 4]. Microbes such as bacteria and fungi in gut insects play a key role in their diversity and evolution, as well as during the insect invasion process [5]. Some bacteria reside in specific cells within the insect body and are known as "endosymbionts", while others are located on the body surface and are known as "ectosymbionts" [6, 7]. However, they are still mostly found in gut, where they act as key regulators of lifestyle diversity in insect hosts [8].

Gut microbiota refers to the general term of all microorganisms inhabiting the host's digestive tract, including viruses, archaea, bacteria, fungi and protozoa, but bacteria are the dominant group of most insect gut microbes [9]. There is a complex interaction between intestinal microorganisms and the host, which has an important impact on the metabolism and physiological activities of the host [8, 10]. It can be attributed to the following aspects: (1) Synthesizing some nutrients that are lacking in natural foods but necessary for the growth and development of host [11, 12]; (2) Secreting digestive enzymes for food digestion; (3) Helping to resist predation by natural enemies or invasion by pathogenic bacteria and improving the host's immunity [13, 14]; (4) affecting the life span, developmental period and reproductive ability of host [15, 16]. Thus, insects are highly dependent on their gut microbiota for survival as well as for the regulation of normal life activities. With the gradual deepening of the research on insect intestinal microorganisms, the diversity of intestinal microorganisms and their physiological functions to the host have been revealed, such as locusts [17], *Spodoparia*spp[18]., and their development potential has attracted more and more attention, and has been gradually applied to agriculture, energy, environmental protection and other important fields [15].

*H. axyridis* is an effective natural enemy insect, which is a strong predator with high adaptability and diverse prey [19]. As well as other predatory ladybird, it has a good control effect on a variety of phloem-sucking pests and *Lepidopteran* larvae, such as aphids, scabies, and phylloxera [20–24]. Therefore, *H. axyridis* has long been used as a biological control method in integrated pest control in orchards, farms and greenhouses [25, 26]. However, there is still a restriction of supply. At present, the artificial propagation of *H. axyridis* using the three-level food chain "fava bean-aphid-*H. axyridis*" requires large space utilization and high feeding cost, which is difficult to achieve industrial production. How to rapidly proliferate larges number of natural enemies of insects in a short time and at a low cost has always been a problem to be solved in the industrial production of natural enemy insects [27, 28]. Breeding natural enemies with artificial diet is an important means to improve biological control [29]. The proposal of replacing natural food with artificial feed provides a solution to realize the feeding and population propagation of natural enemies. Therefore, screening and improving the formula of artificial feed are important to improve the fecundity of ladybeetles and promote industrial production [30]. With the progress of research, artificial diets based on traditional pig liver have been widely used, but compared with aphid feeding, they have disadvantages such as long instar duration, low survival rate, low number of eggs laid and low hatching rate, which have a negative impact on ladybug fecundity [31]. In addition, there are also problems such as complex feed formulation, high cost, low application rate, immature production and storage technology, which are difficult to meet the needs of large-scale production [32]. At present, there is no artificial feed for *H. axyridis* on the market that meet the growth and reproduction needs of its commercial production [33]. In order to solve this problem, previous studies in our laboratory have explored the potential of glucose and trehalose as synergistic substances. The results have showed that both two saccharides have significantly shortened the development period and pre-oviposition period of *H. axyridis,* and have improved the number of eggs laid and hatching rate of *H. axyridis* [34]. However, it still cannot make it close to the state of *H. axyridis* that feeds on aphids.

Studies have found that *Serratia* and *Lactococcus* in gut have regulated the synthesis of information chemicals in reflex bleeding of *H. axyridis* for defensing [35]. In addition, gut microbiota may play an important role in the nutritional ecology of *H. axyridis*. The gut microbiota of ladybird has quickly responded to food changes, which might contribute to the strong adaptability of the ladybird to the environment [36]. Therefore, in view of the effects of different artificial diets on reproduction of *H. axyridis* [34], it is helpful for understanding the relationship between the diversity of gut bacteria and the reproductive ability of *H. axyridis* to understand the gut microbiota composition of *H. axyridis* feeding on different diets.

In a natural ecosystem, various organisms digest and hydrolyze lignocellulose biomass efficiently. For example, termites, they digest lignocellulose biomass with the help of symbiotic microorganisms in their gut [37]. As well as some effective new lignin-degrading and polysaccharide-hydrolyzing bacteria were isolated and identified from wood-feeding termite's guts [38]. High-throughput sequencing technology is one of the key technologies to analyze the diversity, structure, function and evolutionary relationships of insect gut flora [39], and has been widely used in insect studies, such as honeybees [40] and *Drosophila* [41] and termites [42]. In this study, metagenomic sequencing was used to detect the composition and relative abundance of gut bacteria in *H. axyridis* feeding on four different diets (aphids, pig liver-based basic diet, basic diet + glucose, basic diet + trehalose). This study is helpful for screening suitable probiotics to improve the nutrition of artificial diet for *H. axyridis*.

## Results

### β diversity analysis

**Fig. 1:**
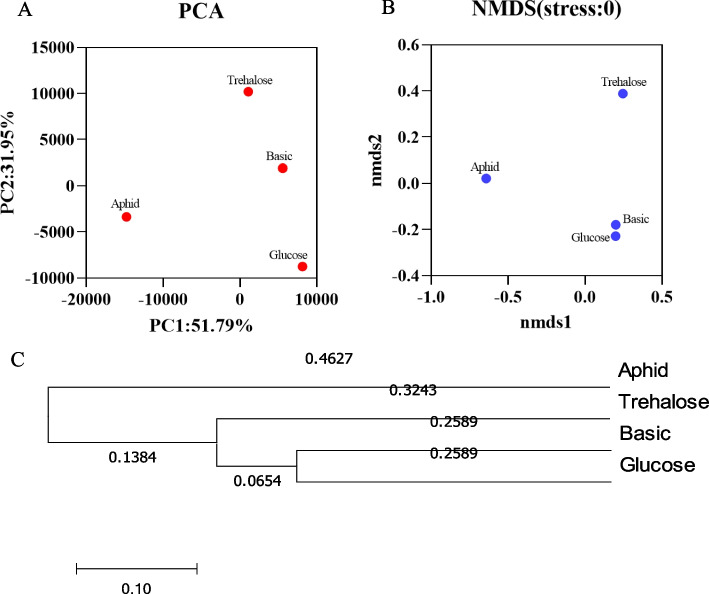
Comparison of β diversity of *Harmonia axyridis* on different diets. **A** *PCA* Principal component analysis, **B** *NMDS* non-metric multidimensional scaling, **C** Hcluster hierarchical clustering tree

The Principal Component Analysis (PCA) and Non-metric Multidimensional Scaling (NMDS) results showed that the distance between the artificial diet groups and the aphid group was relatively far. The results have indicated that the gut microbiota composition of *H. axyridis* feeding on artificial diet and that of feeding on aphid are quite different (Fig. 1A, B). However, the distance between basic group and glucose group was relatively close in NMDS analysis, which suggested that the composition of gut microbiota of *H. axyridis* feeding on these two artificial diets may be relatively similar (Fig. 1B). The hierarchical clustering analysis also showed the basic group and glucose groups were clearly clustered (Fig. 1C).

### Comparison of intestinal microbiota of *H. axyridis* on different diets

**Fig. 2:**
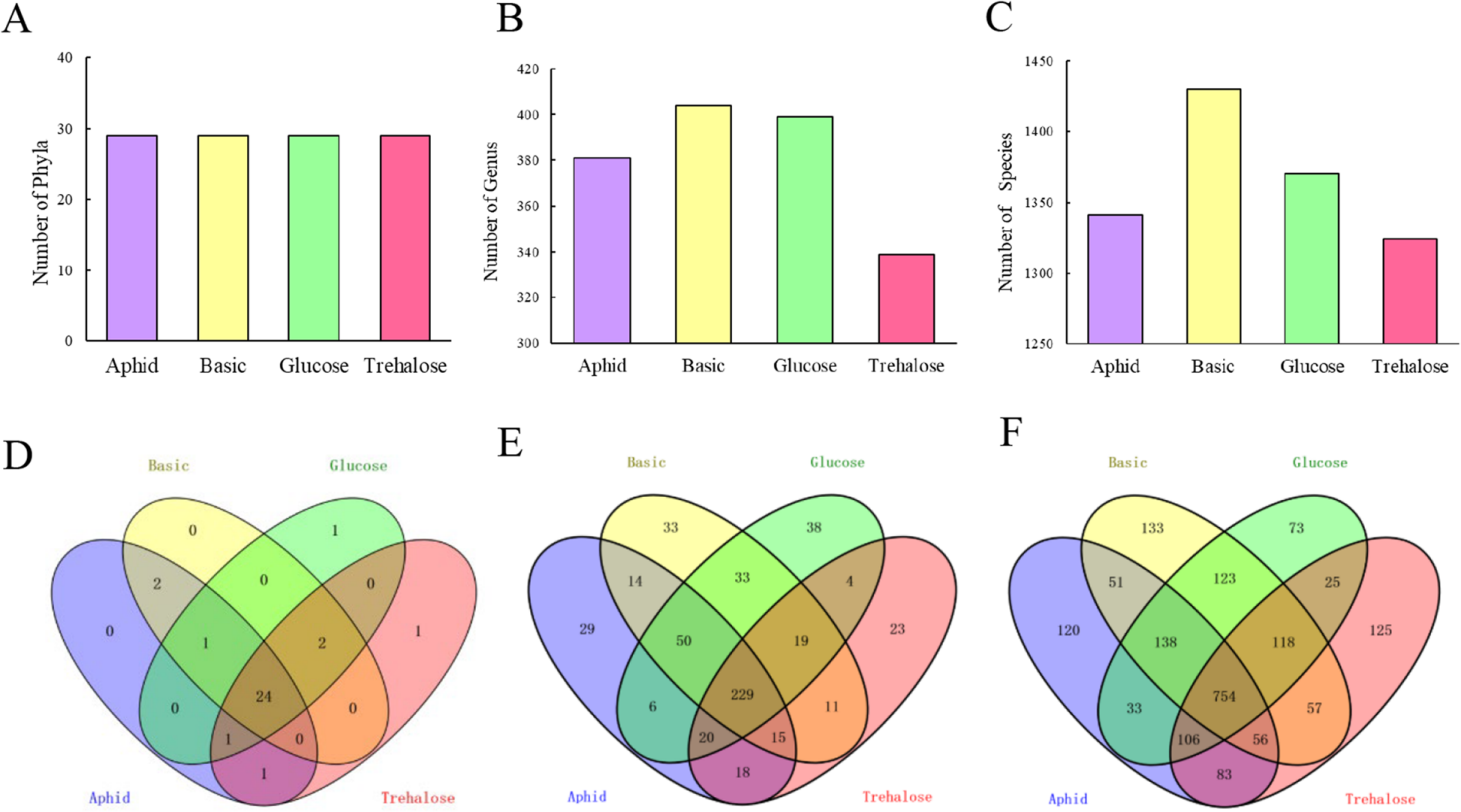
Composition of gut microbiota at phylum, genus and species levels in *Harmonia axyridis* feeding on different diets. Aphid, feeding on aphid; Basic, feeding on basic artificial diet; Glucose: feeding on basic artificial diet supplemented with glucose; Trehalose, feeding on basic artificial diet sup-plemented with trehalose. **A** The difference in the number of Phyla in different feeding groups, **B** The difference in the number of Genus in different feeding groups, **C** The difference in the number of Species in different feeding groups; **D**, **E**, **F**: Phyla, Genus, Species levels Venn diagram)

The results showed that the number of phyla in the gut microbiota of *H. axyridis* feeding on aphids was the same as that of *H. axyridis* feeding on the artificial diets, with 29 phyla (Fig. 2A). However, at the level of genus and species, the gut microbiota of *H. axyridis* in basic group and glucose group were more abundant (Fig. 2B, C).

The gut microbial classes of each group were compared. The results showed that there were 24 phyla shared by four diet group, and there was only one unique phyla in the glucose group and the trehalose group, which was *Cryptophyta* and *Candidatus Saccharibacteria*, respectively (Fig. 2D). In addition, 229 genera were found to be common in all groups, while aphid group, basic group, glucose group and trehalose group had 29, 33, 38 and 23 unique genera, respectively (Fig. 2E). The results of species level analysis showed that the number of common species in the four diet groups was 754, while aphid group, basic group, glucose group and trehalose group had 120, 133, 73, and 125 unique species, respectively (Fig. 2F).

### Comparison of gut microbiota abundance of *H. axyridis* on different diets

**Fig. 3:**
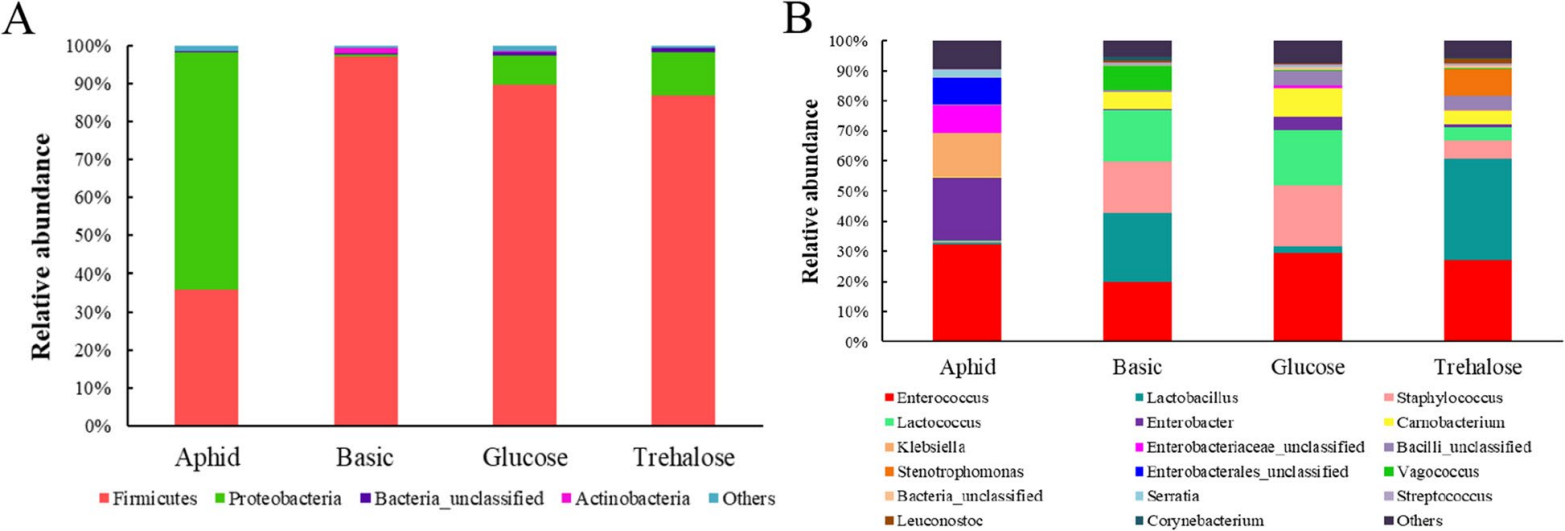
Comparison of the relative abundance of gut microbiota at phylum and genus levels in *Harmonia axyridis* feeding on different diets. (**A** *Firmicutes* and *Proteobacteria* were the major phyla in gut microbiota of *H. axyridis* feeding on aphids, the relative abundance of these two phyla changed greatly in the other groups, **B** The composition of gut microbiota was different among the groups at genus level.)

Results showed that *Firmicutes* and *Proteobacteria* were the major phyla in gut microbiota of *H. axyridis* feeding on aphids, while the relative abundance of these two phyla changed greatly in the other groups. In the basic diet group, the *Firmicutes* proportion (97.09%) was increased compared to aphid group (35.97%). while the *Proteobacteria* proportion decreased (0.6%). However, although the same changes were presented in the glucose and trehalose groups, the proportions of *Firmicutes* in the glucose (89.85%) and trehalose groups (87%) was decreased, and the *Proteobacteria* proportion in the glucose (7.43%) and trehalose groups (11.21%) was increased compared with the basic group, respectively (Fig. 3A). It was suggested that glucose and trehalose alleviated the change. Similarly, the composition of gut microbiota was different among the groups at genus level. The relative abundance of *Enterobacter*, *Klebsiella*, *Enterobacteriaceae*_unclassified, *Enterobacteriales*_unclassified and *Serratia* in the aphid group was higher than that in the artificial diet group, while the relative abundance of *Carnobacterium* was lower than that in the artificial diet group (Fig. 3B). In addition, the relative abundance of *Lactobacillus* in basic group and trehalose group was higher than that in aphid group and glucose group, and the relative abundance of *Staphylococcus* and *Lactococcus* in basic group and glucose group was higher than that in aphid group and trehalose group. The relative abundance of *Stenotrophomonas* was higher in the trehalose group than in the other three groups, while the relative abundance of *Vagococcus* was higher in basic group than in the other three groups (Fig. 3B).

### Annotation of clusters of orthologous groups of proteins (COG) functions

**Fig. 4:**
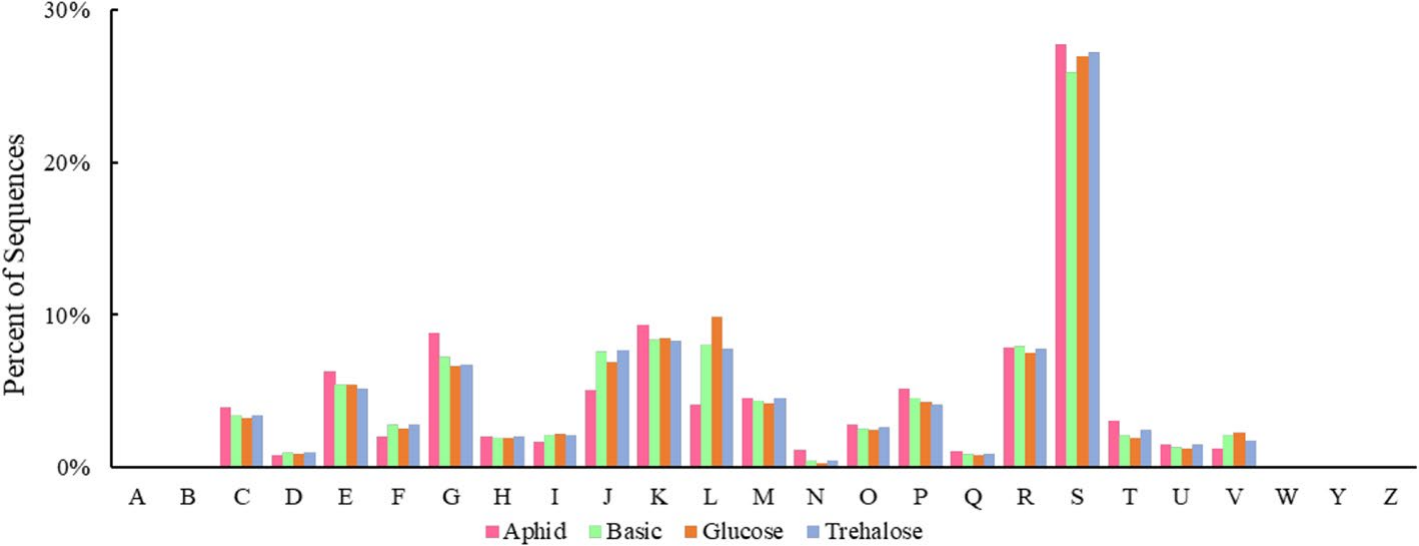
Relative abundance of COG function in intestinal genes of *Harmonia axyridis* feeding on different diets. **A** RNA processing and modification, **B** Chromatin structure and dynamics, **C** Energy production and conversion, **D** Cell cycle control, cell division, chromosome partitioning, **E** Amino acid transport and metabolism, **F** Nucleotide transport and metabolism, **G** Carbohydrate transport and metabolism, **H** Coenzyme transport and metabolism, **I** Lipid transport and me-tabolism, **J** Translation, ribosomal structure and biogenesis, **K** Transcription, **L** Replication, re-combination and repair, **M** Cell wall/membrane/envelope biogenesis, **N** Cell motility, **O** Post-translational modification, protein turnover, chaperones, **P** Inorganic ion transport and metabo-lism, **Q** Secondary metabolites biosynthesis, transport and catabolism, **R** General function pre-diction only, **S** Function unknown, **T** Signal transduction mechanisms, **U** Intracellular trafficking, secretion, and vesicular transport, **V** Defense mechanisms, **W** Extracellular structures, **Y** Nuclear structure, **Z** Cytoskeleton

The sequences with the largest proportion belonged to function unknown and general function prediction only in all groups (Fig. 4). The relative abundance of functions about energy production and conversion (3.92%), amino acid transport and metabolism (6.3%), carbohydrate transport and metabolism (8.8%), transcription (9.4%), inorganic ion transport and metabolism (5.1%), signal transduction mechanism (3.03%), and intracellular transport, secretion and vesicle transport (1.5%) in aphid group were higher than those in artificial diet group (Fig. 4).

### KEGG (Kyoto Encyclopedia of Genes and Genomes) Pathway function annotation

**Fig. 5:**
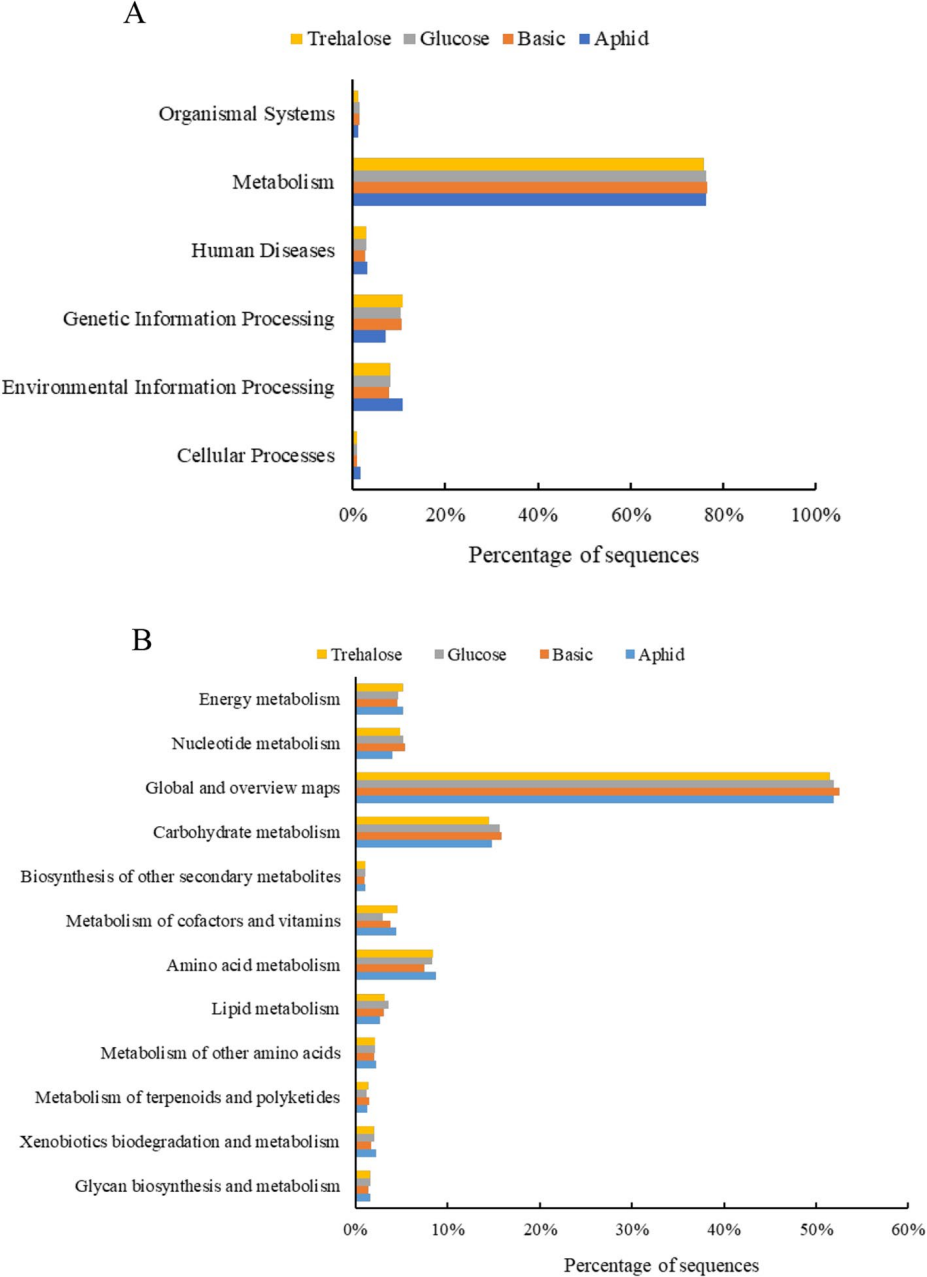
Relative abundance of KEGG pathway (**A**) and metabolism pathway (**B**) in intestinal genes of *Harmonia axyridis* feeding on different diets. Aphid, feeding on aphid; Basic, feeding on basic ar-tificial diet; Glucose: feeding on basic artificial diet supplemented with glucose; Trehalose, feeding on basic artificial diet supplemented with trehalose

Based on the KEGG Pathway annotation, the intestinal genes of *H. axyridis* were divided into organismal systems, metabolism, human diseases, genetic information processing, environmental information processing, and cellular process. Among them, the metabolism pathway accounted for the largest proportion of gene functions in each group (Fig. 5A). In addition, the relative abundance of metabolic pathways enriched by intestinal genes in the glucose group (76.43%) was significantly higher than that in the other three groups, and the relative abundance of metabolic pathways in the aphid group was the lowest (Fig. 5A). Among the metabolism pathways, the carbohydrate metabolism pathway was the most abundant in addition to the global and overview map (Fig. 5B). Besides, the most relative abundance of carbohydrate metabolism pathway presented in glucose group (Fig. 5B).

### Prokaryotic CAZymes in the gut of *Harmonia axyridis*

**Fig. 6:**
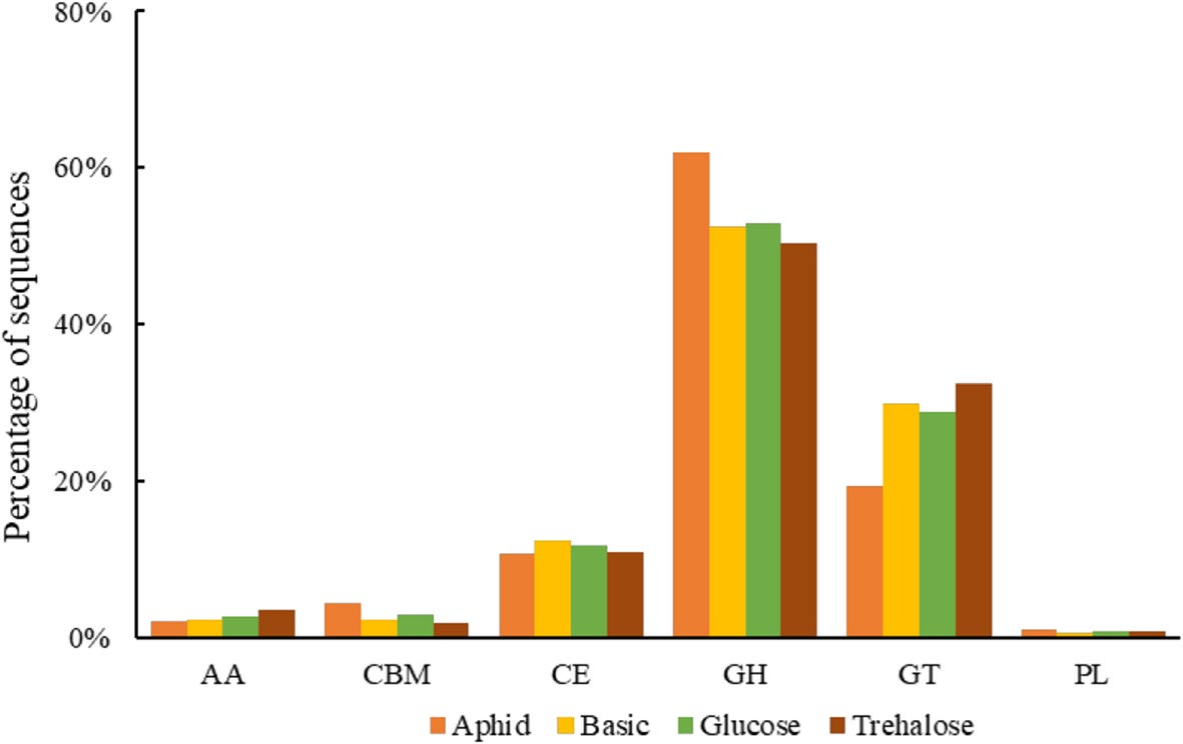
Relative abundance of carbohydrate active enzymes in in intestinal genes of *Harmonia axyridis* feeding on different diets. *AA* Auxiliary activities, *CBM* Carbohydrate-binding modules, *CE* Carbohydrate esterases, *GH* Glycoside hydrolases, *GT* Glycosyltransferases, *PL* Polysaccharide lyases

Results showed that sequences encoding glycoside hydrolases were the most abundant in the intestinal gene of *H. axyridis*, followed by glycosyltransferases (Fig. 6). Notably, the abundance of sequences encoding glycoside hydrolases was much higher in aphid-fed *H. axyridis* (62.06%) intestinal gene compared to artificial diets, but the relative abundance of sequences encoding glycosyltransferases was the lowest among the four diet treatments (Fig. 6).

## Discussion

Nutrition is essential for the complete development and reproduction of *H. axyridis*, and previous studies in our laboratory have shown that diets containing different nutrients have significant effect on the developmental time and reproduction ability of *H axyridis* [34]. Researchers have found that artificial diet feeding patterns affected the composition and structure of intestinal microorganisms in silkworm, and the diversity of intestinal bacteria in silkworm larvae fed with different diets is different [43, 44]. Studies have also reported that diets containing different components affected the intestinal microflora of insects. For example, high-fat diet, high-protein diet and high-starch diet led to significant differences in the intestinal microflora of *Blattella germanica* [45], which may also be one of the reasons why dietary components affect the growth, development and reproduction of insects. Therefore, in this study, the gut microbiota of *H. axyridis* feeding on different diets was determined for exploring the reasons why artificial diets limited the development and reproduction. The results showed there all were 29 phyla in gut microbiota, but the composition and structure were different. The glucose group and the trehalose group had one unique phylum, *Cryptophyta* and Candidatus *Saccharibacteria*, respectively. This indicated that these two phyla might be related to sugar metabolism. In addition, the relative abundance of each phylum was also different. *Proteobacteria* was the most dominant phylum in the aphid group, while *Firmicutes* accounted for the largest proportion in the basic artificial diet group, which was similar to previous studies [46, 47], indicating that artificial diet directly changed the composition and structure of gut microbiota in *H. axyridis*. It was worth noting that the difference caused by basic artificial diet was improved after glucose or trehalose was supplemented in the artificial diet, and the reproduction of *H axyridis* was also increased after glucose or trehalose was added into artificial diet [34]. In addition, *Proteobacteria* was abundant in the intestines and stomach of most vertebrates and participated in various metabolic processes, with important functions in sugar decomposition and fermentation as well as vitamin production [48]. The difference is that members of *Firmicutes* played more important role in protein degradation [49]. Therefore, artificial diet might lead to the reduction of *Proteobacteria* in the gut of *H. axyridis*, thus reducing the utilization of sugar and limiting the development and reproduction of *H. axyridis*. However, the addition of glucose or trehalose could mitigate the limitation of development and reproduction.

The composition of the gut microbiota in the four groups was also quantitatively and structurally different at genus and species level. The gut microbiota of *H. axyridis* on the basic artificial diet and the artificial diet supplemented with glucose were more diverse. The relative abundance of *Enterobacter*, *Klebsiella*, *Enterobacteriaceae*_unclassified, *Enterobacterales*_unclassified, and *Serratia* in the aphid group was higher than that in the artificial diet group. Some species in *Enterobacter* and *Klebsiella* had significant effects on the insect reproduction or lifespan. For example, *Bactrocera dorsalis* female feeding on an artificial diet supplemented with *Enterobacter cloacae* produced more eggs but had a shorter life span, and it was also associated with reproduction in mosquitoes [50, 51]. However, *B. dorsalis* had a longer life span but produced fewer eggs after feeding on the artificial diet supplemented with *Klebsiella oxytoca* [50]. In addition, compared with *Ceratitis rosa *sensu stricto, the relative abundance of *Enterobacter cloacae* and *Klebsiella variicola* is higher in its sister species *Ceratitis quilicii* females, which was more invested in reproduction [52]. These results suggested that *Enterobacter* and *Klebsiella* might be the crucial genera affecting the reproduction of *H. axyridis*.

The gut microbiota of insects may play an important role in the transport and metabolism of carbohydrates, inorganic ions and other nutrients [53]. According to the COG function annotation, it was found that artificial diet reduced the relative abundance of energy production and conversion, amino acid transport and metabolism, carbohydrate transport and metabolism, transcription, inorganic ion transport and metabolism, signal transduction mechanism, and intracellular transport, secretion and vesicle transport in intestinal genes compared with aphid group. Studies on the intestinal tract of honeybees have shown that the gut microbiota promoted host body weight through bacterial metabolism and hormonal signaling [54]. The gut microbiota of locusts has produced phenolic compound, which helped host to improve the resistance to pathogens [55, 56]. The gut microbiota of *Monochamus alternatus* have helped to metabolize sugars and amino acids [57]. Similarly, we have speculated that artificial diet may affect the development and reproduction of *H. axyridis* by inhibiting the metabolic function of gut microbiota, but the mechanism needs to be further studied. However, the enrichment of KEGG metabolic pathways have proved that the relative abundance of metabolic pathways in the aphid group was the lowest in four group, and the reason also needs to be further explored. Notably, the aphid-feeding *H. axyridis* had higher abundance of gut microbiota genes encoding glycoside hydrolases than the other three artificial diets, but the abundance of gut microbiota genes encoding glycosyltransferases was the lowest among the four diet treatments, which may be caused by diet-derived gut microbiota. It was also possible to suggest differences in the absorption of intestinal nutrients in *H. axyridis* after feeding on different foods.

## Conclusions

In conclusion, different diets had obvious effects on the composition and structure of gut microbiota in *H. axyridis*, among which *Proteobacteria*, *Enterobacter* and *Klebsiella* may play a key role. In addition, artificial diet affected the metabolic activity of gut microbiota, which may account for the inhibition of host development and reproduction by artificial diet compared with aphid [46]. The above results have provided a theoretical basis for the screening of synergistic probiotics in artificial diet and upgrading the artificial diet for *H. axyridis*.

## Methods

### Insects rearing

A population of *H. axyridis* that had been raised in laboratory for more than 5 years was used to study. They were raised in an artificial climate chamber with a temperature of 23 ± 2℃, a relative humidity of 68 ± 5%, and a photoperiod of 16L:8D. *Megoura japonica* was used as the food of *H. axyridis*. They were cultured with *Vicia faba* L. under a temperature of 19 ± 1℃, a relative humidity of 70 ± 5%, and photoperiod of 14L:10D. Faba bean seedlings impregnated with aphids were placed in a cage for rearing the *H. axyridis*. The growth of ladybug was observed and aphids were supplied timely.

### Preparation of artificial diet

The basic artificial diet used in this experiment was based on the previous formula and improved [31]. Because trehalose and glucose were found to be effective artificial diet synergetic substances in previous studies, glucose and trehalose were added to the basic artificial diet [34]. The specific diet formulations are shown in Table 1.

**Table 1:** Artificial diet formulation

component	Basic	Glucose	Trehalose
pig liver	100 g/kg	100 g/kg	100 g/kg
honey	10 g/kg	10 g/kg	10 g/kg
royal jelly	5 g/kg	5 g/kg	5 g/kg
vitamin C	10^4^ IU/kg	10^4^ IU/kg	10^4^ IU/kg
sucrose	20 g/kg	20 g/kg	20 g/kg
glucose	/	20 g/kg	/
trehalose	/	/	20 g/kg

### Feeding treatments and intestinal sampling of *H. axyridis*

*H. axyridis* on the third day after emergence were collected and housed in groups of five pairs (female/male ratio = 1:1). They were fed in a 5 cm × 5 cm × 10 cm plastic box with air vent. The inner walls and the bottom of the box were covered with vermiculite and perlite for the activity of ladybeetles and the attachment of eggs. The control group was fed excessive fresh aphids daily, labeled aphid group. In addition, the experimental groups were fed with artificial diet placed in a strip of plastic groove, which was changed once a day.

Females were collected at 5th, 7th, and 12th days after feeding the diet, and the intestinal tracts were isolated from 5 individuals in each time point. Subsequently, the intestinal tracts from the same group at 5th, 7th and 12th days were mixed (5 × 3 samples in each treatment), and each treatment contains one biological repeat. The detailed anatomical steps are as follows: Wings of adults were removed, the body was immersed in 75% ethanol for 1 min, and cleaned in sterilized water for 30 s. Then, the intestinal tracts were dissected in 1 × PBS buffer under microscope (Leica, Germany). All samples were sent to Nextomics Biosciences Company (Wuhan, China) for sequencing.

### Metagenomic sequencing

Genomic DNA was extracted from the collected samples, and the extracted genomic DNA was detected by 1% agarose gel electrophoresis. After the DNA samples were qualified, genomic DNA was fragmented to about 300 bp, and the PE library was constructed using the TruSeq™ DNA Sample Prep Kit (lllumina, USA) [58, 59]. Subsequently, bridge PCR was performed using cBot Truseq PE Cluster Kit v3-cBot-HS (lllumina, USA). Finally, high-throughput sequencing was performed using Illumina's Hiseq.

### Data processing

Paired-end (PE) sequencing was performed using Illumina sequencing technology. Clean reads were obtained from raw data by mass clipping. The number of sequencing reads in different samples are tabulated in Table 2. Megahit Contigs (https://github.com/voutcn/megahit) was used for assembly. METAProdigal (http://prodigal.ornl.gov/) was used for gene prediction of obtained contigs, and the number of ORFs in each group was analyzed. There are many microorganisms (or genes) in common among samples from the same environment, and the changes in the abundance of different genes between samples could reflect the commonalities and differences between samples. Therefore, a non-redundant gene catalog was constructed to describe the overall information of all genes in this type of environment. Gene sequences were predicted and clustering with CD-HIT software (http://www.bioinformatics.org/cd-hit/) (parameters as follows: 95% identity, 90% coverage). The longest gene in each class was used as a representative sequence to construct a non-redundant gene set.

**Table 2:** The number of sequencing reads in different samples

Sample	Raw reads	Raw bases (bp)	Clean reads	Clean bases (bp)
Glucose	45,841,198	6,876,179,700	43,714,994	6,493,956,221
Aphid	47,245,974	7,086,896,100	44,789,064	6,663,397,013
Basic	40,239,500	6,035,925,000	38,159,958	5,656,999,637
Trehalose	40,489,092	6,073,363,800	38,677,756	5,755,983,316

Salmon (https://github.com/COMBINE-lab/salmon) was used to predict the gene abundance. BLASTP (http://blast.ncbi.nlm.nih.gov/Blast.cgi) was used to species annotation by comparing with NR database. Subsequently, the abundances at each taxonomic level were also calculated using gene abundances. In addition, the COG function of annotated genes was analyzed by comparing with eggNOG database. The KEGG functional annotation was analyzed by comparing with KEGG database. The hmmscan (http://hmmer.janelia.org/search/hmmscan) were used to analyze the the carbohydrates annotation information using CAZy database (http://www.cazy.org/).

### Data analysis and plotting

The relative abundance of each microbiota or sequences were calculated and plotted with Microsoft Excel 2016. The beta diversity was plotted with GraphPad Prism 9, and the Venn Diagram was plotted with Draw Venn Diagram (http://bioinformatics.psb.ugent.be/webtools/Venn/).

## Data Availability

The datasets presented in this study can be found in online repositories. The accession number is PRJNA996316.
